# Craniometric Data Supports Demic Diffusion Model for the Spread of Agriculture into Europe

**DOI:** 10.1371/journal.pone.0006747

**Published:** 2009-08-26

**Authors:** Ron Pinhasi, Noreen von Cramon-Taubadel

**Affiliations:** 1 Department of Archaeology, University College Cork, Cork, Ireland; 2 Department of Anthropology, University of Kent, Canterbury, United Kingdom; State University of New York College at Oneonta, United States of America

## Abstract

**Background:**

The spread of agriculture into Europe and the ancestry of the first European farmers have been subjects of debate and controversy among geneticists, archaeologists, linguists and anthropologists. Debates have centred on the extent to which the transition was associated with the active migration of people as opposed to the diffusion of cultural practices. Recent studies have shown that patterns of human cranial shape variation can be employed as a reliable proxy for the neutral genetic relationships of human populations.

**Methodology/Principal Findings:**

Here, we employ measurements of Mesolithic (hunter-gatherers) and Neolithic (farmers) crania from Southwest Asia and Europe to test several alternative population dispersal and hunter-farmer gene-flow models. We base our alternative hypothetical models on a null evolutionary model of isolation-by-geographic and temporal distance. Partial Mantel tests were used to assess the congruence between craniometric distance and each of the geographic model matrices, while controlling for temporal distance. Our results demonstrate that the craniometric data fit a model of continuous dispersal of people (and their genes) from Southwest Asia to Europe significantly better than a null model of cultural diffusion.

**Conclusions/Significance:**

Therefore, this study does not support the assertion that farming in Europe solely involved the adoption of technologies and ideas from Southwest Asia by indigenous Mesolithic hunter-gatherers. Moreover, the results highlight the utility of craniometric data for assessing patterns of past population dispersal and gene flow.

## Introduction

The debate over the origins of agriculture in Europe has mainly centred on two demographic models. The *demic diffusion* model (also known as the *wave of advance*) suggests a progressive dispersal of Southwest (SW) Asian Neolithic farmers into Europe [1]–[3]. This process involved region-specific and variable degrees of admixture between the incoming farmers and the local Mesolithic hunter-gatherers. Alternatively, a *cultural diffusion* model suggests that agricultural knowledge and technologies diffused from SW Asia into Europe but without a significant demographic expansion of SW Asian farmers [4], [5]. Various intermediate scenarios have also been proposed. Some suggest diffusion as the main underlying mechanism involved (e.g. [6]), while others argue that early European agriculture was developed independently by indigenous Mesolithic hunter-gatherer-forager populations with no diffusion of either knowledge or people from the core SW Asian regions [4].

The first mathematical analysis of chronometric archaeological data on early Neolithic European cultures demonstrated a southeast-northwest (SE-NW) temporal cline across Europe [1]. A re-assessment of the wave of advance model using a much larger data set and calculating the probability of various hypothetical centers of agricultural origin provided further support for the observed clinal pattern [7]. While this cline suggested that agriculture spread across Europe in a SE-NW fashion, the archaeological data alone cannot detect whether this is the outcome of a demic diffusion, cultural diffusion, or a palimpsest of complex demographic and historical processes. Subsequent genetic studies of classical allelic markers using principal components analysis (PCA) reported a similar SE-NW clinal pattern observed when plotting the major component of variation [8]. It has been demonstrated that PCA analyses of spatially correlated genetic data can produce highly structured results which are mathematical artifacts that do not necessarily reflect underlying historical migration and dispersal events [9]. However, partial correlations of classical genetic, temporal, and geographic matrices have also found support for the demic diffusion model [10] and hence imply that in this specific case, the clinal pattern is not artefactual but rather produced by demographic historical processes.

Studies of nuclear DNA polymorphisms [11], [12], autosomal microsatellite loci [13] and various sets of DNA markers [14] all confirm a substantial contribution of Southwest Asian populations to the European gene pool and report similar SE-NW clines across Europe. In contrast, analyses of mtDNA haplogroups [15]–[17] suggest a pre-Neolithic coalescence date and a limited contribution of SW Asian Neolithic farmers (∼20%) to the European gene pool. Studies of Y-chromosome markers provide conflicting results. Semino et al. [18] estimate a contribution of ∼22% from SW Asian farmers, which is in close agreement with the figures provided for mtDNA haplogroups. However, a recent reassessment using a different methodological protocol [19] report an average contribution of SW Asian farmers to the European gene pool of between 50 and 65%. Studies of biallelic Y-chromosome polymorphisms report clines that are in agreement with the demic diffusion model [20].

Employing modern European genetics to assess the nature of the spread of farming from SW Asia into Europe is inherently problematic for several reasons: the size and representativeness of modern samples may not match those of the actual populations affected; observed patterns of genetic variation could be explained by several different evolutionary scenarios [21]; any modern gene genealogy may not portray Neolithic and post-Neolithic population history unless founder effects eliminated pre-existing polymorphisms [21], and molecular coalescence dates of geographically patterned lineages do not necessarily correspond to the timing of arrival of a genetic variant in that region [22]. While ancient DNA studies allow for the direct comparison of archaeological and modern populations, they are also limited by constraining factors such as small and unrepresentative samples, limited authenticity methods, and contamination [23]–[25]. The analysis of ancient mtDNA sequences, extracted and amplified from 24 Neolithic central European specimens, points to a genetic discontinuity between these early farmers and current European populations [26]. The discontinuity is attributed to a negligible genetic contribution of Southwest Asian farmers to the modern European gene pool and hence supports the cultural diffusion model. In contrast, a more recent paleogenetic study of Neolithic samples from the Iberian peninsula [27] indicates long-term genetic continuity in this region since the Neolithic. These results suggest a heterogeneous Neolithic dispersal into Europe, which possibly involved acculturation in Central Europe and demic diffusion along the Mediterranean coast [27]. These conflicting results indicate that paleogenetic studies are, as yet, not extensive enough to provide conclusive results regarding the genetic contribution of SW Asian farmers to the European gene pool.

In contrast, the availability of extensive samples of Mesolithic and Neolithic skulls from SW Asia and Europe provide a unique opportunity to assess the biological relationship between pre-farming and early farming populations. In recent years, several studies have demonstrated that human cranial shape variation is largely congruent with an evolutionary model of neutral expectation (e.g., [28]–[38]). Therefore, patterns of human cranial shape covariation can be employed successfully as a proxy for neutral genetic evidence of past population history [34], [39]. Thus, we use this empirical relationship between craniometric and neutral genetic variation to test several alternative hypotheses of population change in Europe associated with the spread of farming from SW Asia.

**Figure 1:**
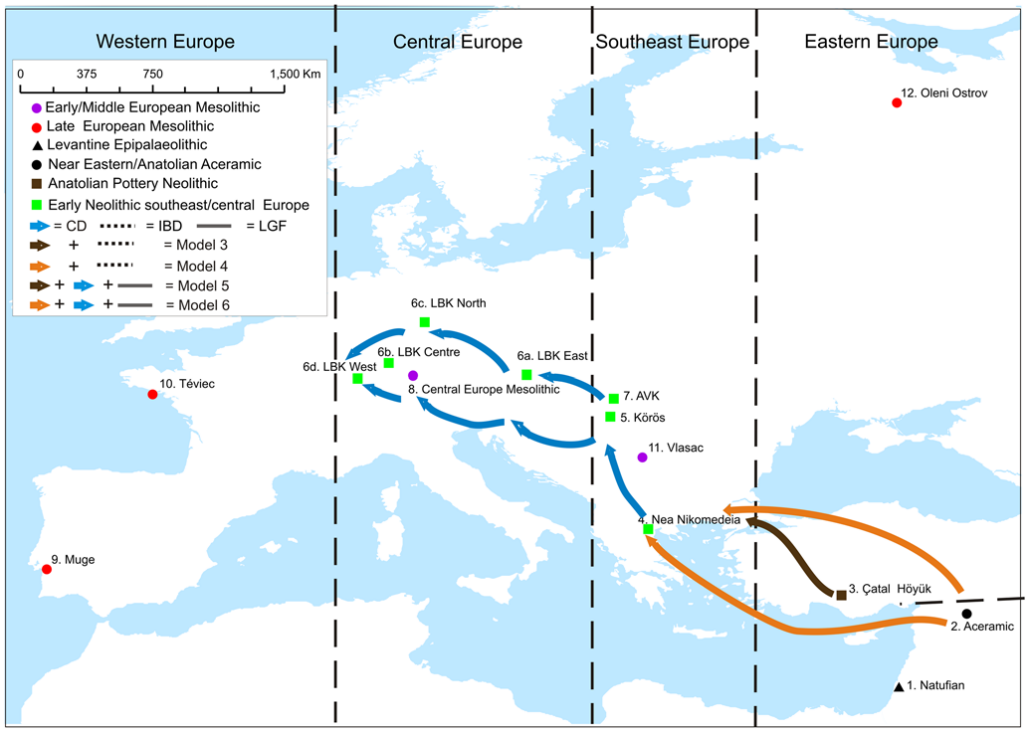
Map showing geographic distribution of all OTUs. Dispersal models involving the active migration of people from SW Asia take two basic forms. Once-off single dispersals from either Anatolia (brown arrow) or the Levant (orange arrows), or continuous dispersal models whereby active population migration continued from southeastern Europe into central Europe (blue arrows). CD = Continuous dispersal, IBD = Isolation-by-distance (null), LGF = Limited gene flow.

**Figure 2:**
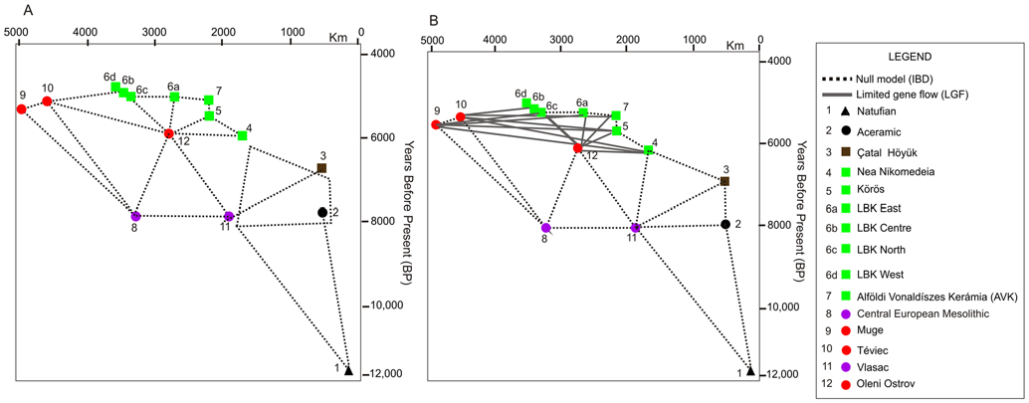
Two-dimensional representation of the null model of isolation-by-geographic and temporal distance. The horizontal axis represents the distance between all OTUs and OTU1 (Natufian) in km. The vertical axis represents the chronological distribution of the OTUs in average years B.P. A. The null model expects all OTUs to be related to each other according to their distribution in space, once time is corrected for (dashed lines). B. The LGF model (solid lines) is a variant of the null model, which expects decreased gene flow between contemporaneous Mesolithic (OTUs 9, 10 and 12) and Neolithic (OTUs 4, 5, 6 and 7) populations as a result of cultural barriers imposed by the adoption of agricultural practices.

Competing hypotheses regarding the nature of the spread of farming were tested using a Mantel matrix correlation approach [40], [41] and an extensive craniometric dataset representing Mesolithic and early Neolithic SW Asian and European populations (Table S1). These OTUs (operational taxonomic units) represent the best available craniometric data for Mesolithic and early Neolithic populations in these regions (Figure 1). In contrast with previous studies of this kind [40], [41], we do not use arbitrary values (0, 0.5, 1 etc.) to quantify the hypothesised morphological distances between OTUs in our alternative model matrices. Rather, we create a null model based upon evolutionary expectations of cranial differentiation according to the principles of isolation-by-geographic and temporal distance [42] (Figure 2). This is based on the observation that there is a strong correspondence between geographic distance and genetic distance in humans for neutrally evolving markers such as microsatellites [33]. Therefore, in the absence of non-neutral forces such as natural selection or directional long-range dispersal, the expected neutral pattern of craniometric diversity would correlate with geographic distance, once the effect of temporal distance is controlled for [42]. In turn, all alternative models are variations of the null model reflecting different hypothesised scenarios (see Materials and Methods). We conduct a series of partial Mantel tests [43] to assess the congruence between craniometric distance and each of the geographic model matrices, while controlling for temporal distance. We particularly chose to focus on the neolithization process in those European regions which preceded the further spread of agriculture into the northern and western parts after 5000 cal. BC [5], [44], [45]. The null model (Figure 2a) reflects the expected pattern of morphological distance between populations if the expansion of farming was largely an indigenous process involving minimal or no dispersal from SW Asia. We then test 5 alternative hypothesized scenarios (models 2 to 6) in order to determine: (1) the extent to which the transition to farming in Europe was the result of demic diffusion of SW Asian farmers; (2) whether the process involved single or continuous dispersals; (3) whether the dispersal origin of the SW Asian farmers was more likely located in the Southern Levant or Anatolia; and (4) the extent to which a demic diffusion process also entailed admixture with indigenous European Late Mesolithic populations. In each model, the distances between OTUs were modified according to the expected variation in gene flow between OTUs (see Methods section). In order to assess the effect of employing different geographic distances, dispersal events (i.e., models 3–6) were each modeled three times; as a decrease in geographic distance between the affected OTUs, of 500, 1000, or 1500 km per migration event. Similarly, limited gene flow (model 2) was modeled as an increase in geographic distance of 500, 1000 or 1500 km between the relevant OTUs (Figure 2b).

## Results

**Figure 3:**
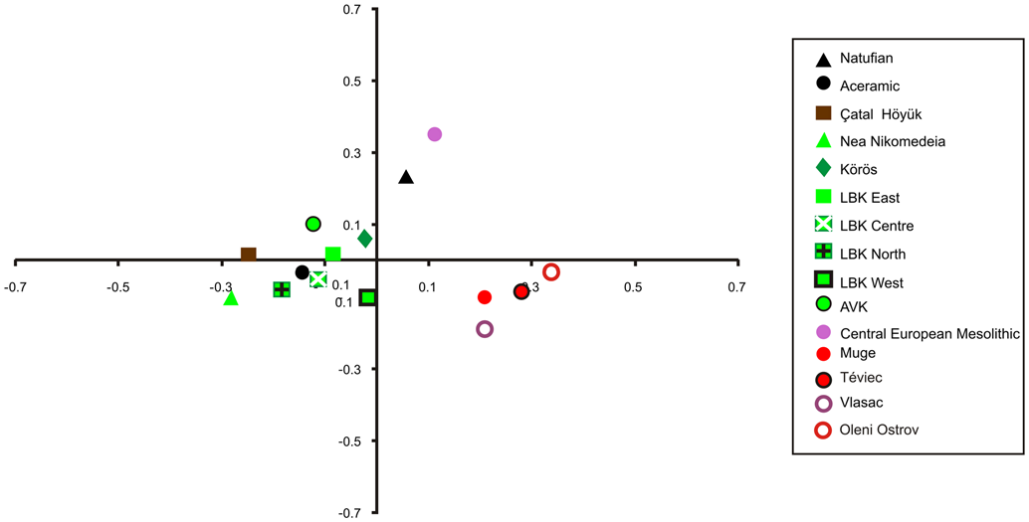
Plot of the first two principal co-ordinates illustrating OTU affinity patterns based on craniometric data. The major axis of variation (horizontal axis, 34.9% variance) shows a clear distinction between all archaeologically defined Neolithic OTUS (green, brown and black circle symbols) and all Mesolithic OTUS (purple and red symbols) plus the Natufian (black triangle). Second axis = 18.7% variance.

Figure 3 plots the first two principal co-ordinates of the craniometric distance matrix. The OTUs do not group according to any particular geographic or temporal pattern on the first or second principal co-ordinates. However, the first principal co-ordinate separates the archaeologically defined Neolithic OTUs from OTUs designated as Mesolithic plus the Natufian. Therefore, the principal co-ordinate analysis suggests that Neolithic and Mesolithic populations are biologically differentiated.

The results of the partial correlations (Table 1) show that the null model was not significantly correlated with the craniometric distance matrix, once temporal variation was controlled for. The null model reflects the degree of differentiation we would expect amongst OTUs under a model of pure cultural diffusion. Of the alternative scenarios modelled, all variants of the continuous dispersal models were significantly correlated with the craniometric data, regardless of whether the dispersals were modelled as originating in Anatolia or in the Levant, and regardless of which geographic parameter was employed to model dispersal (500, 1000 or 1500 km). In contrast, no model of a single dispersal from SW Asia was significantly correlated with the craniometric data. There was some support for a hypothesis of biological change due to a restriction on gene flow between contiguous Mesolithic and Neolithic populations, but only when this barrier was modelled to be relatively strong (i.e., increase of 1000 or 1500 km). These results support a model of demic dispersal from the Near East, as opposed to a model of pure cultural diffusion or sporadic single dispersal events.

**Table 1:** Partial Mantel test results are given as correlation coefficients (r-values) with p-values in parentheses.

Models	Strength of modelled dispersal/gene flow events			
		500 km	1000 km	1500 km
1. Null	0.31 (0.040)			
2. Limited gene flow		0.37 (0.020)	**0.42 (0.015)**	**0.45 (0.005)**
3. Single dispersal from Anatolia		0.34 (0.030)	0.36 (0.030)	0.37 (0.023)
4. Single dispersal from Levant		0.32 (0.027)	0.34 (0.025)	0.36 (0.027)
5. Continuous dispersal from Anatolia		**0.38 (0.017)**	**0.43 (0.011)**	**0.45 (0.010)**
6. Continuous dispersal from Levant		**0.41 (0.014)**	**0.48 (0.003)**	**0.52 (0.002)**

Partial Mantel tests correlate model matrices based on geographic distance and the craniometric distance matrix, while controlling for temporal distance. Significant results, following Bonferroni correction (p≤0.017), are in bold.

The results of the Dow-Cheverud tests (Table 2) mirror those of the partial Mantel tests in showing that the continuous dispersal models and the model of restricted gene flow between Mesolithic and Neolithic populations are significantly more likely than the null model (Table 2, left column). In the comparison of pairs of alternative models, the Dow-Cheverud results also show that there is no statistical difference between dispersal models originating in Anatolia or the Levant (i.e., SD Levant = SD Anatolia; CD Levant = CD Anatolia). The results also show that models of continuous dispersal (i.e., CD Anatolia, CD Levant) fit the craniometric data significantly better than single dispersal models (i.e., SD Anatolia, SD Levant). Moreover, a continuous dispersal model from the Levant is significantly more likely than a model of restricted gene flow between Mesolithic and Neolithic populations when distances of 500 km and 1000 km are assumed (see Table 2, right column).

**Table 2:** The Dow-Cheverud test results (p1Z scores and p-values in parentheses) for comparisons of alternative models against the null model (left column) plus comparisons of several alternative models (right column).

Comparisons with Null	500 km	1000 km	1500 km	Model Comparisons	500 km	1000 km	1500 km
**LGF**	**0.44 (0.002)**	**0.42 (0.006)**	**0.40 (0.002)**	**SD Anatolia/SD Levant**	0.07 (0.370)	0.02 (0.419)	<0.001 (0.49)
**SD Anatolia**	0.26 (0.060)	0.23 (0.070)	0.21 (0.090)	**CD Anatolia/CD Levant**	0.26 (0.059)	0.27 (0.058)	0.25 (0.068)
**SD Levant**	0.25 (0.060)	**0.26 (0.040)**	0.23 (0.060)	**SD Levant/CD Levant**	**0.46 (0.002)**	**0.42 (0.006)**	**0.38 (0.009)**
**CD Anatolia**	**0.47 (<0.001)**	**0.43 (0.002)**	**0.39 (0.005)**	**SD Anatolia/CD Anatolia**	**0.35 (0.008)**	**0.31 (0.027)**	**0.28 (0.044)**
**CD Levant**	**0.54 (<0.001)**	**0.53 (<0.001)**	**0.47 (<0.001)**	**CD Levant/LGF**	**0.27 (0.041)**	**0.22 (0.050)**	0.16 (0.090)

Significant results (p≤0.05) are in bold. LGF = Limited gene flow, SD = Single dispersal, CD = Continuous dispersal.

## Discussion

The results of both the partial Mantel and the Dow-Cheverud tests indicate that the Neolithic and Mesolithic craniometric patterns better fit a model which includes the active dispersal of people (and their genes) from SW Asia, when compared with a neutral model where no such migration(s) occurs. Similarly, modelling the initial spread of farming as once-off single migrations does not explain the craniometric pattern better than the null model of cultural diffusion. Even allowing for a decrease in contact between exogenous farmers and indigenous hunter-gatherers (i.e., the LGF model) does not account for the craniometric affinity pattern as well as a model of continuous dispersal from SE Asia. Currat & Excoffier [46] demonstrated using a series of genetic simulations that even a small percent of genetic admixture between hunters and farmers would result in an extensive amount of pre-Neolithic contribution to the current European gene pool. Our null model of cultural diffusion allows for admixture between Mesolithic and Neolithic populations living contemporaneously under a model of isolation-by-distance. However, the results show that it is more likely that the arrival of farming in Europe was accompanied by the active dispersal of people from SW Asia, which created a barrier to gene flow between hunters and farmers during the period of co-existence. We, therefore, do not rule out some gene flow between hunters and farmers but argue that the craniometric data does not support *strong* admixture between Neolithic and Mesolithic populations.

Our analysis could not resolve the question of the origin of the dispersal process or its most probable timing. A previous craniometric study [47] indicates that its centre of origin may have been central Anatolia, while spatial interpolations of the radiocarbon chronometric Neolithic record suggest a centre of origin in the Levant [7]. However, both regions fall within the primary core region in which agricultural societies first emerged more than three millennia prior to the emergence of farming in SE Europe [48], [49]. Recent archaeological research suggests that the diffusion of agriculture from the core region into SE Europe occurred in several waves. The earliest occurred during the early pre-Pottery Neolithic B (EPPNB) circ. cal. 8550-8150 BC, and involved the maritime colonization of Cyprus [50], and possibly Crete and the Peloponnese [51]. A probable second dispersal is associated with the appearance and diffusion of the Fikirtepe Culture (cal. BC 6450- 5900 [52]), which is characterized by dark surfaced monochrome pottery in sites in western Anatolia, eastern Thrace, and possibly further west into the Balkans [49]. A third dispersal occurred a few centuries later and involved the appearance of burnished red pottery, the use of anthropomorphic and zoomorphic vessels, clay figurines, and other stylistic characteristics. During this dispersal hundreds of new Neolithic sites appear in western Anatolia and the Balkans [49]. Despite the overall uniformity in artefacts and domesticated crops and livestock which were part of the “Neolithic package”, archaeological research reveals some stylistic variations suggesting continuous endemic population movements and trade within and between these regions [53]. These movements would have involved extensive gene flow along the dispersal route from Anatolia to SE Europe.

In conclusion, our results indicate that the craniometric data best fit a model of continuous demic diffusion into SE and Central Europe from the SE Asian core region in which agricultural societies first emerged. These results are in agreement with most genetic studies which report a considerable genetic contribution of SW Asian farmers to the modern European gene pool [6], [14], [18], [19], [22], [46]. We found no strong support for significant admixture between contemporaneous Mesolithic and Neolithic populations, or for an indigenous adoption of agriculture by Mesolithic populations as has been proposed by some archaeologists (e.g., [5], [54]) and geneticists (e.g., [16], [26], [55]).

Our results illustrate the utility of craniometric data for assessing past population history and highlight the importance of testing hypotheses within a population genetics framework. Our study does not deny or contradict models that propose the existence of regional variability in Europe. In the northern and northwestern regions of Europe the transition to agriculture was possibly more complex and gradual, entailing a larger degree of genetic contribution from indigenous populations [47], [56]. In fact, the evidence amassed from a number of regional case studies indicate that the Neolithic transition process probably involved various demographic and biogeographic mechanisms such as leapfrog colonization, jump dispersals, range expansions, and folk migrations (cf. various contributions in [57]). Future model-bound and simulation analyses of craniometric and genetic data from a wider geographic range will shed more light on these issues. However, our results do not support the assertion that the initial spread of agriculture into Europe proceeded purely as a cultural diffusion event, but instead involved the active dispersal of people from SW Asia.

## Materials and Methods

### Samples

We utilise a set of 116 Mesolithic/Epipalaeolithic and 165 Early Neolithic crania from SW Asia and Europe. These were divided into 15 operational taxonomic units [OTUs] (cf. [41], Figure 1, Table S1), each comprising at least 10 individuals. These OTUs represent samples from biological populations that are defined according to their archaeological, spatial and temporal contexts (Table S1). In all cases, an OTU comprises specimens from a single major archaeological phase. Whenever possible, we construct OTUs using specimens from a single site (e.g., Çatal Höyük, Oleni Ostrov) or specimens from a well defined phase in a given region (e.g., Linienbandkeramik (LBK) East). Sampling was constrained by uneven spatial, temporal and archaeological representativeness of certain phases – for example the Aceramic (Pre-Pottery) sample from the Near East – yet this dataset comprises the best available cranial samples whose archaeological contexts and skeletal preservation facilitate their inclusion in these OTUs. The 15 OTUs provide a secure dataset for testing the contribution of SW Asian early Neolithic farmers to the southeastern and central European gene pool.

### Craniometric distance matrix

The craniometric data comprised 15 standard calliper measurements (see Table S2) taken on samples of skulls representing each of the 15 OTUs described in Table S1. Given the fragmentary nature of many of these archaeological specimens, some of the data were missing from the initial database. Only individual skulls with data present for at least 70% (i.e., 10 measurements) of all measurements were included in the analysis [58]. Missing data were estimated in *SPSS v.16*, within-sexes and within-OTUs, using a multiple linear regression algorithm. These data were adjusted for individual differences in isometric scaling by dividing each cranial variable by the geometric mean of all variables for that individual [59], [60]. Craniometric distance matrices were generated in *RMET 5.0*, software written by John Relethford to perform population genetics analysis using quantitative phenotypic traits [61]). Hence, multivariate biological D-matrices (based on a phenotypic analogue of Wright's [62] F_st_) were calculated under the assumption that population phenotypic variances are proportional to genetic variances [61], [63].

### Constructing model matrices

The isolation-by-geographic distance model predicts a positive relationship between increased genetic (and phenotypic) population differentiation and geographic distance [42], [64]. However, the effect of temporal distance between archaeological OTUs is more difficult to model. There is empirical evidence to suggest temporal autocorrelation for craniometric data, implying a positive relationship between temporal distance and craniometric distance within individual archaeological sites [65]. However, the exact parameters of a model of isolation-by-time *and* space are currently unclear. Given these uncertainties, we base our null model on geographic distance only and employ partial correlations to control for the effects of temporal distance [42]. The null model matrix was subsequently altered to reflect five alternative scenarios of migration and/or restricted gene flow, based on competing hypotheses of whether the spread of farming proceeded primarily as a migration of people (genes) or of culture (Figure 1):

1. Null: The null model is the expected pattern of morphological distance between OTUs if the expansion of farming was largely an acculturation event (Figure 2a).
2. LGF (limited gene flow): As in the null model, farming expands under the parameters of cultural but not genetic diffusion, but this cultural shift causes biological change due to a restriction of gene flow between culturally ‘Neolithic’ and ‘Mesolithic’ OTUs living contemporaneously in Europe (Figure 2b).
3. SD Anatolia (Single dispersal from Anatolia): There was a single human migration from Anatolia (Çatal Höyük) into southeastern Europe, followed by cultural diffusion into central Europe (Figure 1).
4. SD Levant (Single dispersal from Levant): There was a single human migration of Aceramic populations (Levant) into southeastern Europe, followed by cultural diffusion into central Europe (Figure 1).
5. CD Anatolia (Continuous dispersal from Anatolia): There was a human migration from Anatolia (Çatal Höyük) into southeastern Europe, followed by further human migrations into central Europe (Figure 1).
6. CD Levant (Continuous dispersal from Levant): There was a human migration from the Levant into southeastern Europe, followed by further human migrations into central Europe (Figure 1).

Geographic distances between all OTUs were calculated in kilometres as great circle distances based on the haversine [66], [67]. Hence, the distance (*D*) between two points specified by latitudinal (*α*_1_, *δ*_1_) and longitudinal (*α*_2_, *δ*_2_) co-ordinates with a central angle of *θ* between the two points was computed as:
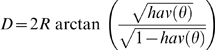
Where *hav* (*θ*) = sin^2^ 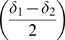 +cos δ_1_ cos δ_2_ sin^2^ 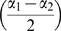 and *R* = radius of earth (6,371 km).

As several OTUs were comprised of pooled specimens from different sites (Table S1), all pairwise geographic distances were rounded to the nearest 500 km. Where several sites were combined to create a single OTU, the average geographic coordinate was taken. Temporal distances were calculated, on a pairwise basis between all OTUs, as the square root of the squared differences between chronological estimates provided in Table S1. Table S3 provides the geographic and temporal distance matrices employed in the partial Mantel tests.

In order to tests the hypothetical scenarios described in the text, the null model was altered to create seven alternative model matrices, reflecting different migration and/or gene flow processes. The LGF model differs from the null model in assuming that the adoption of Neolithic practices by some European OTUs (4, 5, 6 and 7) caused a barrier to gene flow between these populations and contemporaneous un-acculturated Mesolithic populations (9, 10 and 12). Therefore, the biological distances between OTUs (4–7 and 9, 10 and 12) are modelled as lengthened by either 500, 1000 or 1500 km compared with the ‘null’ distances. In the Single Dispersal (SD) models, farming is spread by a once-off migration of people from Anatolia (OTU 3) or the Levant (OTU2) into Europe thereby decreasing the distances between these OTUs and OTUs 4–7 by either 500, 1000 or 1500 km. The Continuous Dispersal (CD) models hypothesises the spread of farming to result from the dispersal of people from Anatolia (OTU 3) or the Levant (OTU2) into Europe and then the repeated dispersal of people from SE to Central Europe according to Figure 2. This results in a decrease in distance of either 500, 1000 or 1500 km between all pairs of OTUs 3,4,5,6 and 7.

### Statistical Analyses

The correlations between the craniometric, geographic and temporal matrices were compared statistically using Mantel matrix correlations [68]. As with a standard Pearson correlation, coefficients (r-values) range from −1 (perfect negative correlation) to +1 (perfect positive correlation). However, because matrix elements cannot be considered independent, significance (p-values) was assigned through a randomisation test, where the observed matrix correlation was assessed against a distribution of correlations obtained by 9999 random permutations of the rows and columns of the matrices [43], [69]. The basic Mantel test allows for the comparison of two matrices (X and Y). However, a partial Mantel test can be performed to control for a third matrix (Z). This is achieved by regressing the elements of X and Y onto Z, and using the residuals from the regressions as the input for a standard Mantel test [43]. Here, we employed partial Mantel correlations to assess the fit of the craniometric data to the six alternative models based on a null model of geographic distance, while correcting for temporal distance. Bonferroni correction was applied to the partial Mantel tests, thereby lowering the accepted α-level to 0.017 [34], [37]. Partial Mantel tests were performed in *PASSaGE 1.1*, freely available from Michael Rosenberg (www.passagesoftware.net).

In order to determine whether or not any of the six alternative model matrices differed significantly in their congruence with the craniometric distance matrix, a series of Dow-Cheverud [70] tests were performed. Here, the null hypothesis is that the correlation of A (craniometric distance matrix) and B (Model 1) is equal to the correlation of A and C (Model 2). If the null hypothesis is rejected (p≤0.05), then one model is significantly more likely than the other. In order to control for temporal distance, each of the model matrices and the craniometric distance matrix were regressed onto the temporal distance matrix, and the resultant residual matrices were used as input for the Dow-Cheverud tests. All comparisons were performed in *R*, employing a code written by the lab of C.C. Roseman. As discussed in detail by Konigsberg [71], all matrix comparisons (Mantel and Dow-Cheverud tests) assume that the biological affinity matrices are known without error. Given that the biological matrices employed here were generated from relatively small samples (10–31 crania per OTU) there is an error inherent to the estimation of the biological relationships between OTUs. Therefore, we add the caveat that all significance values associated with Mantel and Dow-Cheverud tests reported here are minimum values.

## Supporting Information

Table S1 Archaeological samples employed to construct the operational taxonomic units (OTUs).
(0.12 MB DOC)

Table S2 Description and codes of craniometric variables employed
(0.03 MB DOC)

Table S3 Null model matrices of geographic distance (km; lower triangle) and temporal distance (years; upper triangle).
(0.06 MB DOC)
