# Supplementary material for: Craniometric Data Supports Demic Diffusion Model for the Spread of Agriculture into Europe
Source: PLoS One. 2009 Aug 26;4(8):e6747. doi: 10.1371/journal.pone.0006747 (PMC2727056; doi:10.1371/journal.pone.0006747)
Supplement: Table S1 — Archaeological samples employed to construct the operational taxonomic units (OTUs). (0.12 MB DOC) [file pone.0006747.s001.doc]

Table S1. Archaeological samples employed to construct the operational taxonomic units (OTUs).

| **OTU** | **Name** | **Lat** | **Long** | **Site** | **Location** | **Arch. Period** | **Av. Date BC** | **M** | **F** | **U** | **Total N** |
| --- | --- | --- | --- | --- | --- | --- | --- | --- | --- | --- | --- |
| **1** | Natufian | 32.76 | 35.36 | Ain-Mallaha (Eynan) | S Levant | Natufian | 11500 | 2 |  |  |  |
|  |  | 31.42 | 35.06 | Erq-El-Ahmar | S Levant | Natufian | 11500 | 2 |  |  |  |
|  |  | 32.67 | 35.00 | Nahal Oren | S Levant | Natufian | 11500 | 5 | 1 |  |  |
|  |  | 32.90 | 35.22 | Hayonim | S Levant | Natufian | 11500 | 2 |  |  | **12** |
| **2** | Aceramic | 35.87 | 38.40 | Abu Hureyra | Syria | Aceramic (PPNB) | 8,000 | 2 | 2 | 1 |  |
|  |  | 30.23 | 35.53 | Basta | Jordan | Aceramic (PPNB) | 8,000 | 1 |  |  |  |
|  |  | 38.23 | 39.65 | Çayönü | Anatolia | Aceramic | 8,000 | 3 | 1 |  | **10** |
| **3** | Çatal Höyük | 37.10 | 32.13 | Çatal Höyük | Anatolia | Pottery Neolithic | 6900 | 8 | 8 |  | **16** |
| **4** | Nea Nikomedia | 40.65 | 22.30 | Nea Nikomedia | Greece | Early Neolithic | 6150 | 4 | 8 | 1 | **13** |
| **5** | Körös | 46.22 | 20.25 | Deszk-Olajkut | SE Europe | Körös | 5700 | 1 | 1 |  |  |
|  |  | 46.94 | 20.78 | Endröd | SE Europe | Körös | 5700 | 1 |  |  |  |
|  |  | 46.36 | 20.18 | Hódmezövásárhely | SE Europe | Körös | 5700 | 1 |  |  |  |
|  |  | 46.41 | 20.31 | Kotacpart | SE Europe | Körös | 5700 | 3 | 3 | 1 | **11** |
| **6a** | LBK East | 48.66 | 16.59 | Kleinhadersdorf | C-E Europe | LBK East | 5200 | 4 | 1 |  |  |
|  |  | 47.43 | 16.23 | Poettsching | C-E Europe | LBK East | 5200 | 1 |  |  |  |
|  |  | 48.42 | 16.36 | Poysdorf | C-E Europe | LBK East | 5200 | 1 |  |  |  |
|  |  | 49.00 | 14.00 | Radonice-Louny | C-E Europe | LBK East | 5200 | 2 |  |  |  |
|  |  | 49.00 | 16.18 | Rybniky | C-E Europe | LBK East | 5200 | 1 |  |  |  |
|  |  | 48.75 | 16.47 | Schletz | C-E Europe | LBK East | 5200 | 2 | 1 |  |  |
|  |  | 47.47 | 18.42 | Sturovo | C-E Europe | LBK East | 5200 |  | 1 |  | **14** |
| **6b** | LBK Centre | 49.38 | 8.58 | Schwetzingen | Central Europe | LBK Centre | 5200 | 8 | 4 |  |  |
|  |  | 48.85 | 9.21 | Viesenhäuser Hof | Central Europe | LBK Centre | 5200 | 11 | 8 |  | **31** |
| **6c** | LBK North | 50.93 | 10.98 | Bischleben | C-N Europe | LBK North | 5200 | 4 | 2 |  |  |
|  |  | 51.18 | 10.78 | Bruchstedt | C-N Europe | LBK North | 5200 | 2 | 8 |  |  |
|  |  | 52.15 | 11.22 | Eilsleben | C-N Europe | LBK North | 5200 | 1 |  |  |  |
|  |  | 51.03 | 10.85 | Klienfahner | C-N Europe | LBK North | 5200 |  |  | 1 |  |
|  |  | 51.18 | 11.12 | Seehausen | C-N Europe | LBK North | 5200 | 1 | 1 |  |  |
|  |  | 51.12 | 10.54 | Sondershausen | C-N Europe | LBK North | 5200 | 5 | 3 |  |  |
|  |  | 51.82 | 11.93 | Wulfen | C-N Europe | LBK North | 5200 |  | 1 |  | **29** |
| **6d** | LBK West | 48.30 | 7.30 | Entzheim | Western Europe | LBK West | 5000 | 1 |  |  |  |
|  |  | 48.36 | 7.42 | Hoenheim | Western Europe | LBK West | 5000 | 6 | 2 | 1 |  |
|  |  | 49.98 | 8.07 | Niederingelheim | Western Europe | LBK West | 5000 | 1 |  |  |  |
|  |  | 48.36 | 7.30 | Oberschaeffolsheim | Western Europe | LBK West | 5000 |  | 1 |  |  |
|  |  | 47.42 | 7.30 | Rixheim | Western Europe | LBK West | 5000 | 1 | 2 |  | **15** |
| **7** | AVK | 46.70 | 21.26 | Békés-Povád | SE Europe | Szakálhát | 5300 | 2 | 1 |  |  |
|  |  | 46.40 | 20.31 | Hódmezövásárhely-Gorsza | SE Europe | Szakálhát | 5300 | 1 |  |  |  |
|  |  | 47.50 | 20.50 | Kisköre-Gát | SE Europe | Szakálhát | 5300 | 2 |  |  |  |
|  |  | 46.64 | 21.32 | Gyula | SE Europe | Late AVK | 5300 | 1 |  |  |  |
|  |  | 46.94 | 20.23 | Vésztő-Mágori halom | SE Europe | Late AVK | 5300 | 6 | 1 |  | **14** |
| **8** | Central Europe Mesolithic | 48.30 | 10.00 | Hohelnstein | Central Europe | Mesolithic | 8000 | 1 | 1 |  |  |
|  |  | 48.48 | 10.30 | Möttingen-Lierheim | Central Europe | Mesolithic | 8000 | 1 |  |  |  |
|  |  | 48.48 | 10.30 | Nördlingen-Holheim | Central Europe | Mesolithic | 8000 | 7 | 4 |  | **14** |
| **9** | Muge | 39.06 | -8.42 | Muge Arruda | Portugal | Late Mesolithic | 5500 | 1 | 2 |  |  |
|  |  | 38.37 | -8.58 | Muge Moita De Sebastiao | Portugal | Late Mesolithic | 5500 | 3 | 5 |  | **11** |
| **10** | Téviec | 47.51 | -3.15 | Téviec (Frayer) | Atlantic Europe | Late Mesolithic | 5300 | 6 | 7 |  |  |
|  |  | 47.33 | -2.87 | Hoedic | Atlantic Europe | Late Mesolithic | 5300 | 2 | 2 |  | **17** |
| **11** | Vlasac | 44.31 | 22.01 | Vlasac | SE Europe | Mesolithic | 8870 | 9 | 11 |  | **20** |
| **12** | Oleni Ostrov | 62.42 | 35.00 | Oleni Ostrov | Russia | Late Mesolithic | 6100 | 19 | 9 |  | **28** |
| **Total** |  |  |  |  |  |  |  |  |  |  | **255** |
