# Supplementary material for: Craniometric Data Supports Demic Diffusion Model for the Spread of Agriculture into Europe
Source: PLoS One. 2009 Aug 26;4(8):e6747. doi: 10.1371/journal.pone.0006747 (PMC2727056; doi:10.1371/journal.pone.0006747)
Supplement: Table S2 — Description and codes of craniometric variables employed (0.03 MB DOC) [file pone.0006747.s002.doc]

Table S2. Description and codes of craniometric variables employed

| **Measurement** | Howells [66] | Martin and Saller [67] |
| --- | --- | --- |
| Maximum vault length | GOL | M1 |
| Maximum vault breadth (at parietals) | XCB | M8 |
| Minimum frontal breadth | XFB | M9 |
| Maximum frontal breadth | WFB | M10 |
| Bi-auricular breadth | AUB | M11b |
| Bi-asterionic breadth | ASB | M12 |
| Basion-bregma height | BBH | M17 |
| Basion-prosthion length | BPL | M40 |
| Basion-nasion length | BNL | M5 |
| Bizygomatic breadth | ZYB | M45 |
| Orbital breadth | OBB | M51a |
| Orbital Height | OBH | M52 |
| Upper facial height (nasion-prosthion) | NPH | M48 |
| Nasal breath | NLB | M54 |
| Nasal height | NLH | M55 |
