# Supplementary material for: Craniometric Data Supports Demic Diffusion Model for the Spread of Agriculture into Europe
Source: PLoS One. 2009 Aug 26;4(8):e6747. doi: 10.1371/journal.pone.0006747 (PMC2727056; doi:10.1371/journal.pone.0006747)
Supplement: Table S3 — Null model matrices of geographic distance (km; lower triangle) and temporal distance (years; upper triangle). (0.06 MB DOC) [file pone.0006747.s003.doc]

Table S3. Null model matrices of geographic distance (km; lower triangle) and temporal distance (years; upper triangle).

| **Temporal (yrs)** | 1. Natufian | 2. Aceramic | 3. Çatal Höyük | 4. Nea Nikomedeia | 5. Körös | 6a. LBK East | 6b. LBK Centre | 6c. LBK North | 6d. LBK West | 7. AVK | 8. Central Eur Meso | 9. Muge | 10. Téviec | 11.Vlasac | 12. Oleni Ostrov |
| --- | --- | --- | --- | --- | --- | --- | --- | --- | --- | --- | --- | --- | --- | --- | --- |
| **Geographic (km)** |
| 1. Natufian | 0 | 3500 | 4600 | 5350 | 5800 | 6300 | 6300 | 6300 | 6500 | 6200 | 3500 | 6000 | 6200 | 3500 | 5400 |
| 2. Aceramic | 512 | 0 | 1100 | 1850 | 2300 | 2800 | 2800 | 2800 | 3000 | 2700 | 0 | 2500 | 2700 | 0 | 1900 |
| 3. Çatal Höyük | 538 | 725 | 0 | 750 | 1200 | 1700 | 1700 | 1700 | 1900 | 1600 | 1100 | 1400 | 1600 | 1100 | 800 |
| 4. Nea Nikomedeia | 1629 | 1862 | 1148 | 0 | 450 | 950 | 950 | 950 | 1150 | 850 | 1850 | 650 | 850 | 1850 | 50 |
| 5. Körös | 2134 | 2255 | 1606 | 636 | 0 | 500 | 500 | 500 | 700 | 400 | 2300 | 200 | 400 | 2300 | 400 |
| 6a. LBK East | 2648 | 2778 | 2125 | 1077 | 523 | 0 | 0 | 0 | 200 | 100 | 2800 | 300 | 100 | 2800 | 900 |
| 6b. LBK Centre | 3358 | 3536 | 2855 | 1733 | 1295 | 792 | 0 | 0 | 200 | 100 | 2800 | 300 | 100 | 2800 | 900 |
| 6c. LBK North | 3291 | 3424 | 2771 | 1694 | 1170 | 648 | 310 | 0 | 200 | 100 | 2800 | 300 | 100 | 2800 | 900 |
| 6d. LBK West | 3473 | 3672 | 2981 | 1845 | 1449 | 961 | 192 | 494 | 0 | 300 | 3000 | 500 | 300 | 3000 | 1100 |
| 7. AVK | 2146 | 2248 | 1615 | 693 | 84 | 535 | 1321 | 1177 | 1481 | 0 | 2700 | 200 | 0 | 2700 | 800 |
| 8. Central Eur Meso | 3200 | 3379 | 2697 | 1576 | 1141 | 646 | 158 | 302 | 316 | 1169 | 0 | 2500 | 2700 | 0 | 1900 |
| 9. Muge | 4894 | 5248 | 4525 | 3436 | 3323 | 2947 | 2267 | 2566 | 2076 | 3382 | 2359 | 0 | 200 | 2500 | 600 |
| 10. Téviec | 4524 | 4778 | 4066 | 2918 | 2606 | 2133 | 1351 | 1601 | 1172 | 2644 | 1487 | 1157 | 0 | 2700 | 800 |
| 11.Vlasac | 1854 | 2000 | 1332 | 378 | 288 | 794 | 1537 | 1440 | 1677 | 326 | 1379 | 3454 | 2811 | 0 | 1900 |
| 12. Oleni Ostrov | 2711 | 2356 | 2361 | 2541 | 2262 | 2520 | 3198 | 2919 | 3390 | 2183 | 3094 | 5451 | 4513 | 2277 | 0 |
